# CuI *p*-type thin films for highly transparent thermoelectric *p-n* modules

**DOI:** 10.1038/s41598-018-25106-3

**Published:** 2018-05-02

**Authors:** Bruno Miguel Morais Faustino, Diogo Gomes, Jaime Faria, Taneli Juntunen, Guilherme Gaspar, Catarina Bianchi, António Almeida, Ana Marques, Ilkka Tittonen, Isabel Ferreira

**Affiliations:** 1 CENIMAT/I3N, Departamento de Ciência dos Materiais, Faculdade de Ciências e Tecnologia, Universidade Nova de Lisboa, Caparica, 2829-516 Portugal; 2 Department of Electronics and Nanoengineering, Aalto University, P.O. Box 13500, FI-00076 Aalto, Finland

## Abstract

Developments in thermoelectric (TE) transparent *p*-type materials are scarce and do not follow the trend of the corresponding *n*-type materials – a limitation of the current transparent thermoelectric devices. *P*-type thermoelectric thin films of CuI have been developed by three different methods in order to maximise optical transparency (>70% in the visible range), electrical (σ = 1.1 × 10^4^ Sm^−1^) and thermoelectric properties (ZT = 0.22 at 300 K). These have been applied in the first planar fully transparent *p-n* type TE modules where gallium-doped zinc oxide (GZO) thin films were used as the *n*-type element and indium thin oxide (ITO) thin films as electrodes. A thorough study of power output in single elements and *p*-*n* modules electrically connected in series and thermally connected in parallel is inclosed. This configuration allows for a whole range of highly transparent thermoelectric applications.

## Introduction

Wide band-gap metal oxide semiconductors are often used in transparent applications, such as photodetectors, solar cells, light-emitting diodes, *etc*. Despite of their potential in novel applications in the field of energy harvesting, *p*-type transparent semiconductors usually lack the level of performance required in both transparency and electrical conductivity. TE devices are no exception as most established TE materials tend to be optically opaque due to their small band-gap.

Thermoelectric properties are usually assessed via a dimensionless figure of merit in which the larger the ZT value the higher the thermoelectric conversion efficiency:

1 $${\rm{ZT}}={{\rm{\sigma }}{\rm{S}}}^{2}{\rm{T}}/{\rm{k}}$$

where S is the Seebeck coefficient, σ the electrical conductivity, T the temperature and k thermal conductivity. As such, a high value of S and low value of k is desired, adding to a wide band-gap for optically transparent electronics. Transparent *n*-type TCOs with thermoelectric potential, often Al-doped ZnO^1^ or Gallium-doped ZnO^2^, are abundant and in constant optimization. However, *p*-type transparent TCOs with good thermoelectric properties are still far from matching *n*-type materials in combining high transparency with high electrical conductivity. In a recent review of *p*-type oxides, Zhang *et al*.^3^ highlighted thermoelectric properties of major *p*-type oxides in literature and provided a comparison with Sn:InO_2_ (ITO). In general, *p*-type oxides when exhibiting high transparency have also a very low electrical conductivity, whilst less transparent materials often show the contrary. La_0.97_Sr_0.03_CuOS is perhaps the oxide which, to date, best comprises a high transmittance and a high conductivity^4^. The evolving *p*-type TCOs have been considered towards further delocalisation of the valence band to enhance orbital hybridization between O *2p* and the metal cations, where Cu *3d*^10^, Ni *3d*^8^, Cr 3*d*^*3*^ based oxide materials have been the focus^5^.

Nevertheless, CuI is not an oxide but is one of the most simultaneously transparent and conductive *p*-type material and has been highly reconsidered recently. The first report on CuI was in 1907, where Bädeker reported CuI films with a conductivity of 20 S/cm and high transparency after the iodisation of 200–300 nm Cu films in iodine vapour^6^. Its transparency to visible light is conferred by the wide energy gap. In fact, copper (I) iodide in its γ phase (γ-CuI) shows a direct bandgap of 3.1 eV and a *p*-type carrier conductivity due to an acceptor level above the valence band introduced by the copper vacancies^7^. Because of the smaller electronegativity of iodine compared to oxygen, and the valence band edge made up of copper 3*d* and iodine 5*p* states, the electron hole density is much higher on CuI than in Cu(*I*) oxides^7^. These characteristics resulted in today’s interest in CuI and its application in the field of optoelectronics, photocatalytic water splitting, electrode contacts in organic solar cells and OLEDs, and recently in CuI/Al-doped ZnO heterojunctions^8^ – allowing further introduction of CuI thin films in transparent electronics^3^. Thermoelectric generators (TEGs) made of CuI have also been considered, including recent reports of CuI thin films on a flexible substrates^9–11^, but to date only single-module-thermoelectric studies have been reported and are not fully transparent.

In this study, highly conductivity *p*-type γ-CuI thin films were produced and applied to fully transparent *p-n* TE modules electrically connected in series. A thorough study of the power output performances is compared with well-known TE bulk materials. Moreover, prior to the application of CuI in thin film TEGs, three different processes for *p*-type γ-CuI thin film production were compared and their thermoelectric and optical properties optimised. These were vapour and solid phase iodination of Cu films obtained by resistive thermal evaporation in controlled thickness, and by thermal evaporation of CuI powder.

## Results

Structural, morphological and optoelectronic properties of *p*-type CuI thin films fabricated by thermal evaporation of CuI powder, vapour iodination of Cu substrate, and solid iodination of a Cu substrate, were fully characterized prior to their TEG application.

### Structure and morpholog**y**

**Figure 1:**
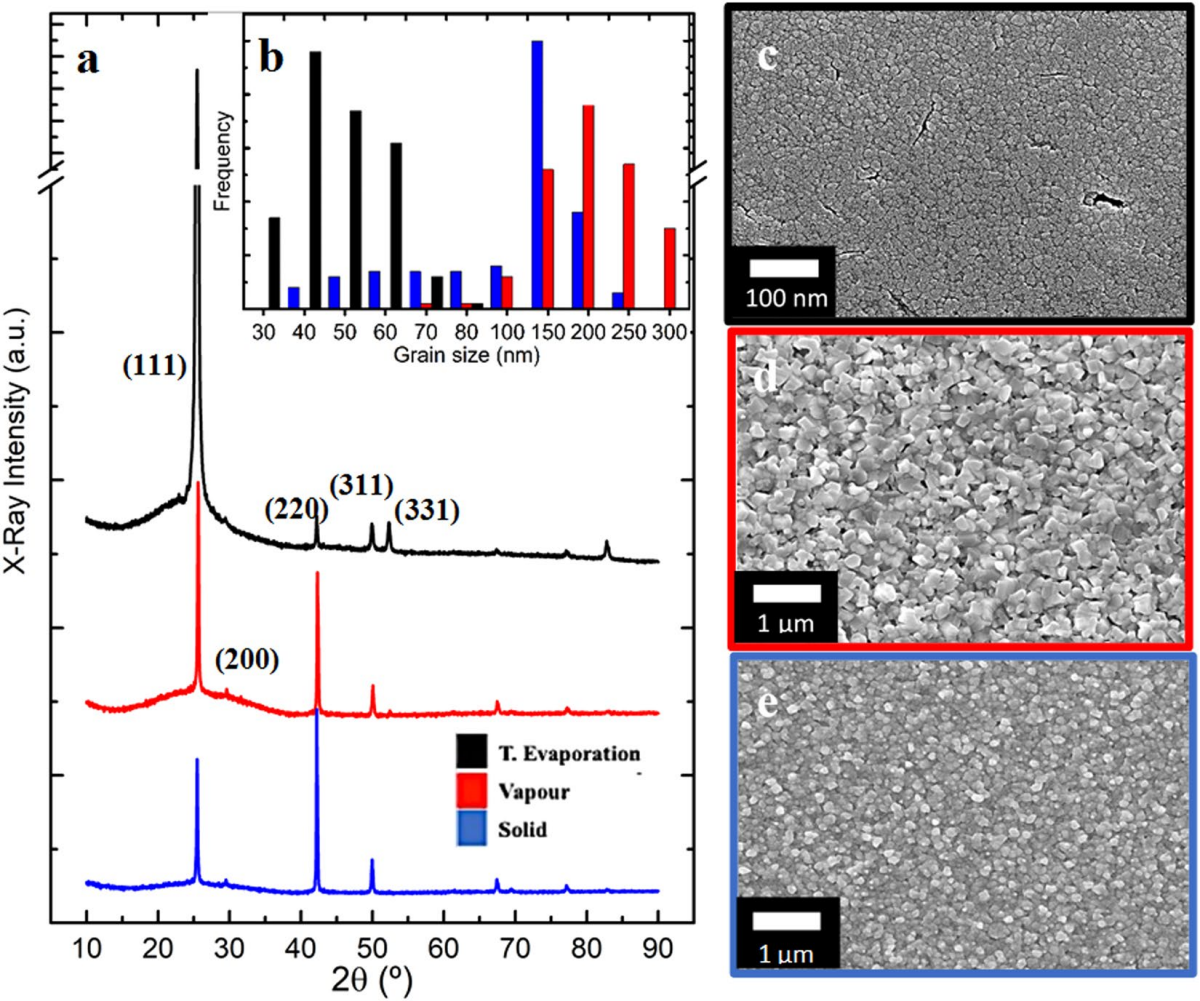
(**a**) X-Ray Diffraction (XRD) spectra and SEM images of CuI obtained by three methods: (**c**) Thermal evaporated; (**d**) Vapour iodination; and (**e**) Solid iodination. A histogram with grain size distribution is presented (**b**).

The crystallography of CuI thin films obtained by each different method is compared in the diffractograms of Fig. 1. All samples show X-ray diffraction patterns in accordance with the literature – diffraction peaks at 25°, 30°, 42°, 50° and 67° correspond to (111), (200), (220), (311), and (331) planes of zinc-blend structure^12^, respectively. Whilst the thermal evaporation route seems to favour the (111) plane, the vapour iodination method reveals the (220) in addition to the main (111) peak. Solid iodination films have a preferential (220) plane and the (111) is much lower in intensity when compared to the previous methods. All peaks are sharp revealing a high level of crystallinity. No other phases were identified, such as the ones for CuO or Cu_2_O, indicating none or undetectable copper oxidation^13^.

**Figure 2:**
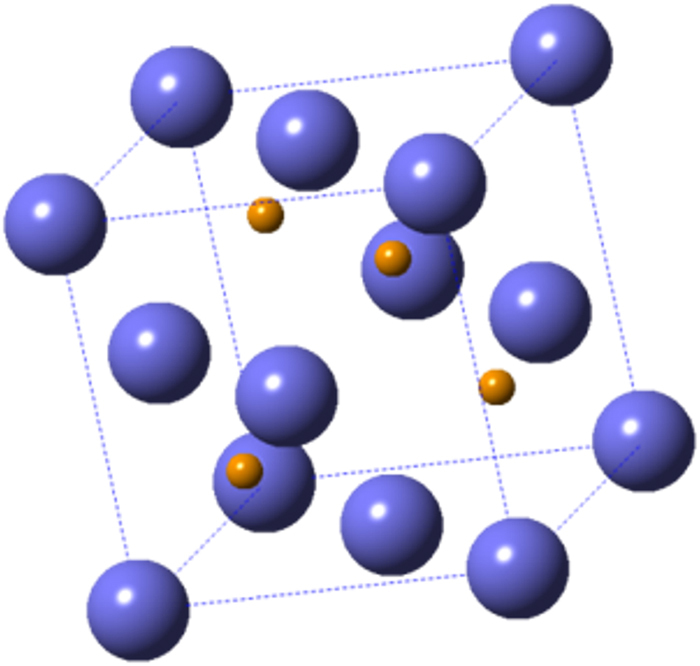
${F}\bar{4}3{m}\,$where a = 0.599 nm and Cu is spatially distributed in octahedral interstitial positions.

The calculated crystallite size via the Scherrer’s formula considering FWHM (Full Width at Half Maximum) of the most intense diffraction plane (111) is 38.7, 49.5 and 48.1 nm for thermal evaporation, vapour and solid samples, respectively (Table 1). For solid phase samples the (220) plane is of higher intensity so the corresponding crystallite size equals 58.2 nm. Average grain sizes were determined from SEM micrographs using the software ImageJ to give 44 nm, 186 nm and 112 nm for thermal evaporation, vapour and solid iodination, respectively. The expected unit cell of γ-CuI calculated from XRD data is represented in Fig. 2, where Cu cations assume the ¼, ¼, ¼ position of the cubic face centred unit cell whose lattice parameter is a = 0.599 nm zinc-blend type with Cu at the centre of a tetrahedron and I anions at vertices. This tetrahedron is formed by one I atom of cubic vertices and the three closer ones at cubic faces, in agreement with literature^14^.

**Table 1:** Crystallite (CS) and grain (GS) sizes obtained by the three methods used. Lattice strain values were calculated considering peak broadening due to microstrain (ε) as: B(2θ) = 4ε (sinθ/cosθ) using the FWHM^25^.

Method	Solid	Vapour	T. Evap
Optimised Thickness nm	287	302	50
CS (111)/nm	48.1	49.5	38.7
*Lattice strain*	0.025	0.036	0.042
CS (220)/nm	58.2	39.5	—
*Lattice strain*	0.018	0.025	—
GS (SEM)/nm	112	186	44
a = b = c (Å)	5.99	5.96	5.99

Surface morphology has proven to be greatly dependent of the iodination process. CuI films by vapour iodination show a higher degree of roughness and larger grain sizes compared to solid films whose grains are smaller and thus revealing less surface roughness. Grain size distribution is broader for solid where grain sizes vary from 30 to 250 nm, whereas in vapour iodination and thermal evaporation methods a narrower grain size distribution is observed.

**Figure 3:**
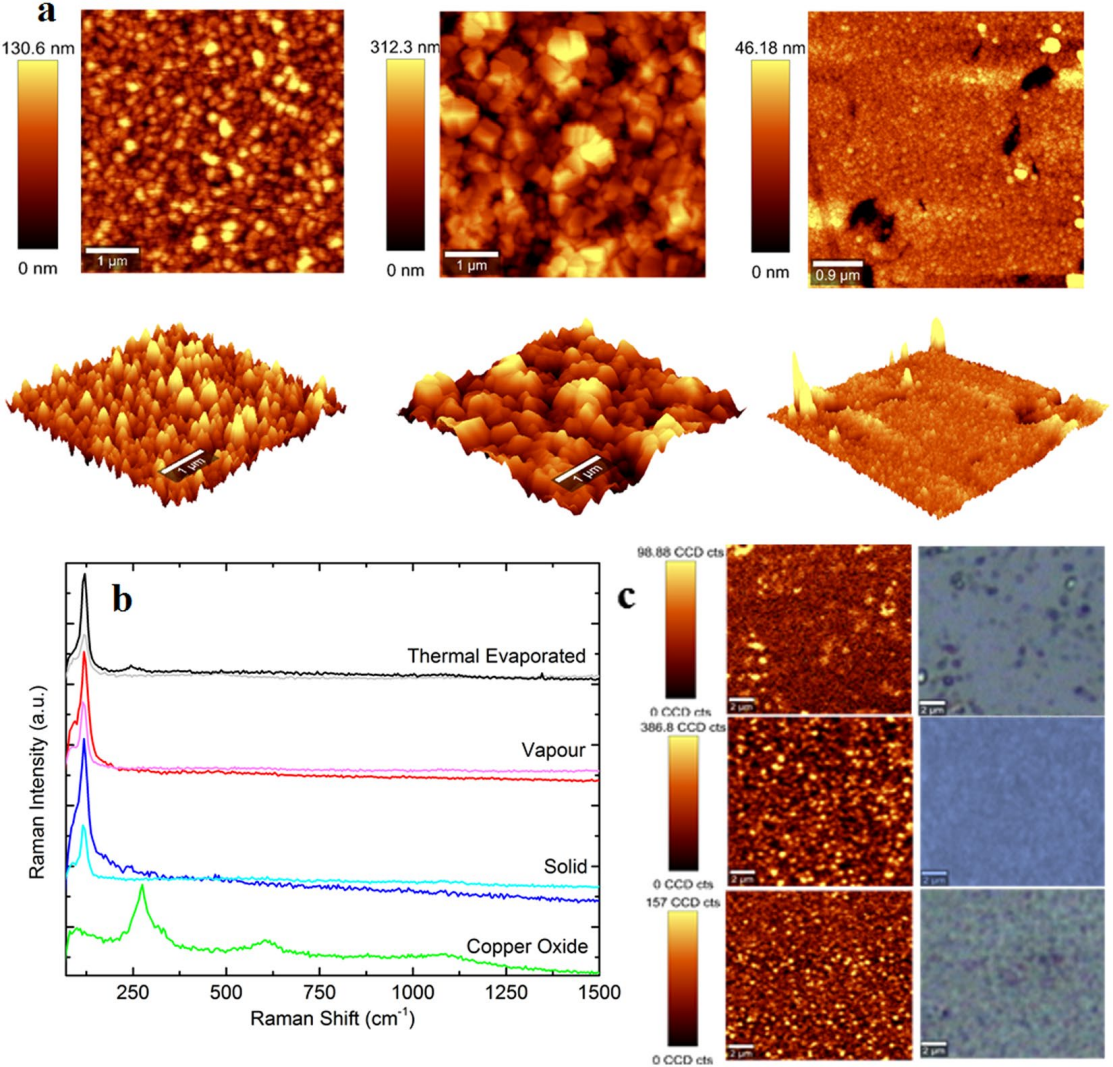
A comparison between methods: (**a**) AFM images: Top – 2D top view, Bottom – 3D side view. (**b**) Normalised Raman intensity, including that of copper oxide. Thinner lines (also brighter in colour) represent samples after 4 months storage; (**c**) Raman spectroscopy mapping whilst monitoring the 120 cm^−1^ peak, all images are at a magnification of ×100.

The AFM microscopy provided a further insight into film topography. The roughness RMS values were calculated to be 83.8 nm, 120.1 nm and 34.4 nm for solid, vapour and thermal evaporation methods, respectively (Fig. 3). The topographic maps and its 3D representation evidences a columnar like structure of CuI films as the dark to bright regions correspond to a height variation of the order of magnitude of the film thickness. The vapour method discloses a higher surface-to-depth ratio with augmented columnar-like structures at full height.

Raman spectroscopy mapping reveals that CuI thin films are highly uniform in terms of composition for all methods. A peak at 120 cm^−1^ is assigned to the optical transverse mode of Cu-I bonding vibration in CuI films according to literature^15^. The CuO and Cu_2_O Raman spectrum differentiates copper iodination versus oxidation to eliminate any uncertainty in regard to oxidation during preparation and storage under room atmosphere and temperature. Indeed, no oxidation peaks (Fig. 3b) are detected up to a period of 4 months in storage at room atmospheric conditions.

### Optoelectronic properties

**Figure 4:**
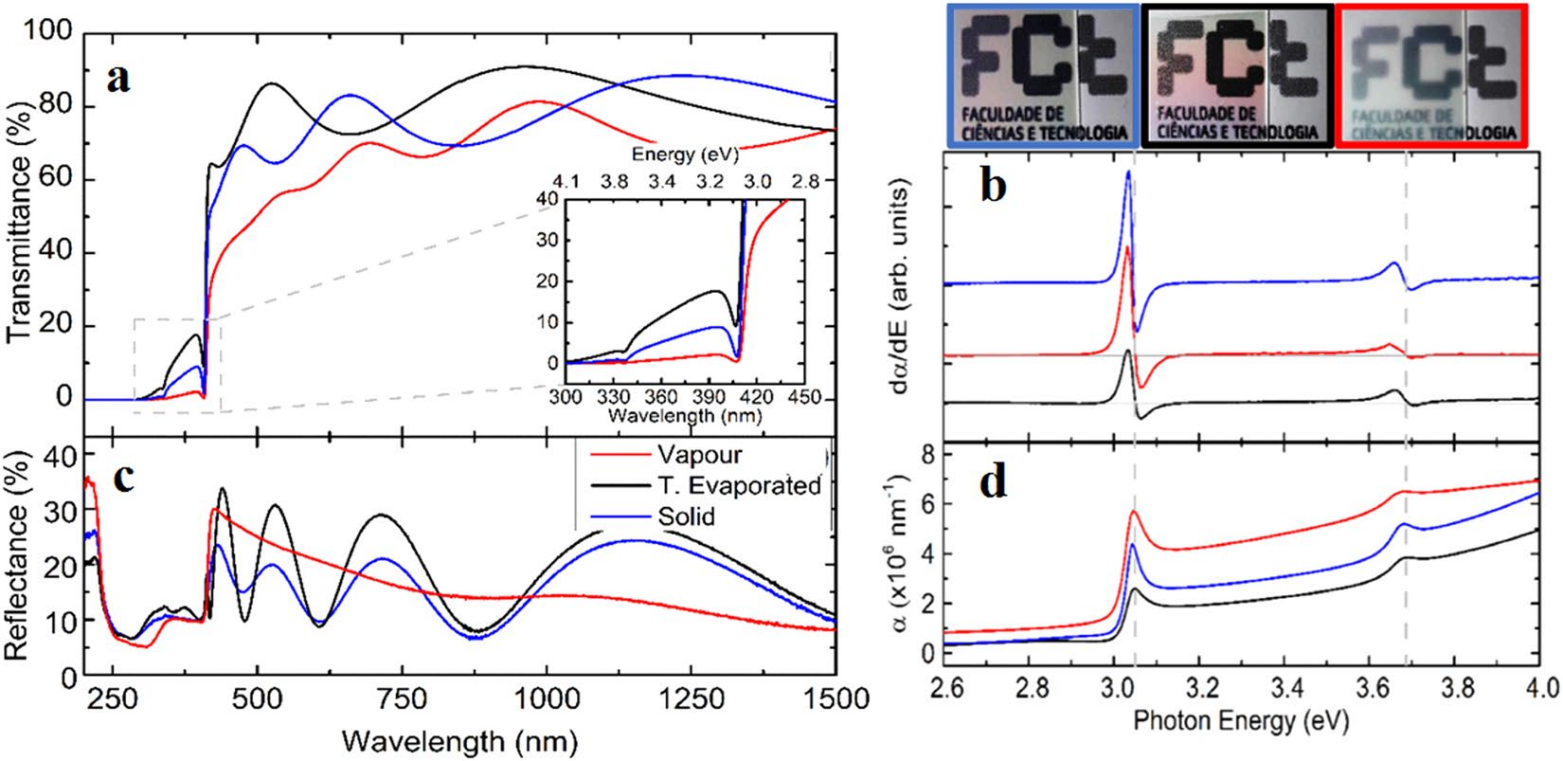
(**a**) and (**c**) a comparison of specular transmittance and total reflectance spectra of CuI films prepared by three methods: vapour (302 nm thick); t. evaporated (50 nm thick); solid (287 nm thick), with an inset (**b**) corresponding to a zoom-in at 300 to 450 nm range. (**b**) the derivative of absorption coefficient to determine the optical band gap and (**d**) absorption coefficient for the band gap energies.

All CuI samples have a very high transmittance with a considerable absorption above 3 eV as revealed by UV-Vis-NIR transmittance and reflectance spectra (Fig. 4). The transmittance in the visible range is generally above 70%, except for the vapour iodination method where samples reveal a lower transmittance value > 50%. Visually, these samples show a frosted-glass like appearance. Interference effects between 400–1500 nm are related to constructive and destructive interferences between light reflected from the film and the substrate interface, resulting in a periodic pattern of peaks as a function of wavelength. The film thickness can be estimated in the transparent region of the spectra applying the Swanepoel method^16^.

CuI is a direct semiconductor material with a band edge absorption at 3.08–3.10 eV and a split off band^5^ at circa 0.6 eV above the valence band edge. This split off band is observed for all samples (Fig. 4).

Further systematic optical optimisation of CuI thin films varying initial Cu thickness for each iodination method and varying iodine vapour exposure times for the vapour method is reported in Supplementary Figure 1.

Aside the optical optimisation, Seebeck coefficients and electrical conductivities were additionally measured. For instance, resistance and PF values were studied as a function of iodination exposure time in vapour method, with a maximum PF value at 2 h iodination time (Supplementary Figure 2); whilst thickness control showed only small variations in the value of Seebeck for samples prepared by thermal evaporation (Supplementary Table 1). As a result, we concluded that samples prepared by solid iodination of Cu thin films were ultimately performing better in both thermoelectric and optical characteristics. A more extensive summary of the thermoelectric properties for all methods is reported in Supplementary Table 4. Consequently, we have selected the solid iodination method for the fabrication of further CuI films to be applied in TE modules. It is an easy and scalable technique leading to films with a high conductivity of 1.1×10^4^ Sm^−1^, a power factor of 4.7×10^−4^ Wm^−1^ K^−2^, a mobility of circa 4.1 cm^2^ V^−1^ s^−1^ of *p*-type carriers with a hole concentration of around 1.7×10^20^ cm^−3^. All electrical measurements, including Hall-effect, were performed at room temperature and atmospheric conditions.

### A transparent TE *p*-*n* module

As in most thermoelectric generators, a *p-n* module is generally preferred to benefit from two different carrier types (*n*-type vs *p*-type material) to maximise thermoelectric potentials of the device/module. These are often connected electrically in series and thermally in parallel, proving very efficient and increasing versatility for applications including refrigeration.

**Figure 5:**
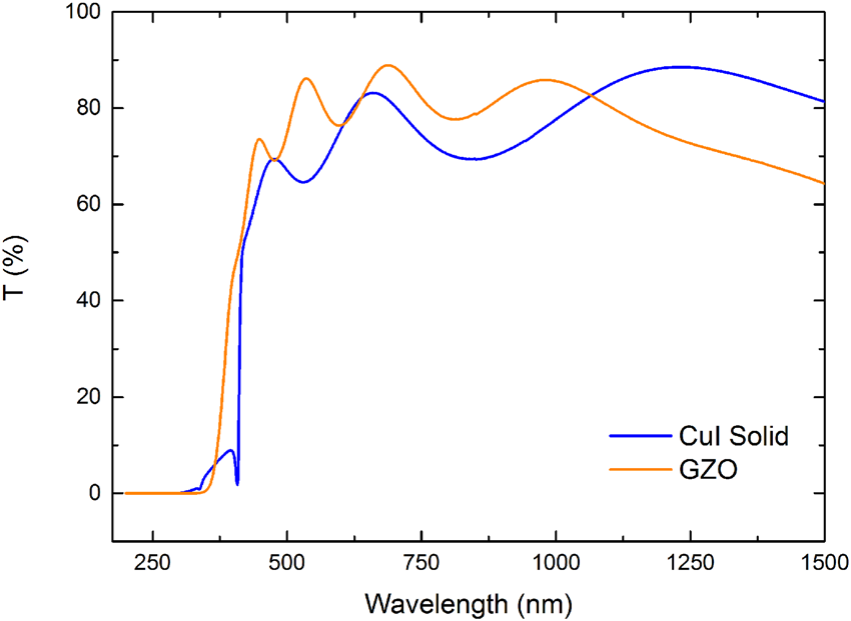
Transmittance spectra of CuI and GZO films produced by the solid iodination and sputtering methods, respectively.

In this study, thin films of gallium-doped zinc oxide (GZO, 361 nm) were chosen as an *n*-type material with a PF 5.3×10^−4^ Wm^−1^ K^−2^, a bulk concentration of 5.9×10^20^ cm^−3^, a mobility of 15 cm^2^ V^−1^s^−1^, an electrical conductivity of 1.42×10^5^ Sm^−1^, and optical properties comparable to those of CuI. Figure 5 compares GZO thin film transmittance to that of CuI thin film by solid iodination. Supplementary Figure 3 illustrates GZO sputtering optimisation with constant working pressure and RF power, varying only film deposition time and its effect on Seebeck coefficients, electrical conductivity and optical transmittance.

Being equally transparent, both CuI and GZO allowed us to build the first fully transparent *p-n* thermoelectric module interconnected with indium tin oxide (ITO, 150 nm, 80 Ω) as a transparent electrode at a distance of 1 cm. The process of TEG modules fabrication is detailed in the Experimental Details section.

To evaluate power output performances, current-voltage (I-V) and power-voltage (P-V) characteristic curves were recorded varying the load resistance and temperature gradient in three configurations: single GZO and CuI as individual elements; and in single and double *p-n* modules (Fig. 5). Indeed, the open circuit voltage V_oc_, the short circuit current I_sc_ and the maximum output voltage V_out_ of single elements GZO and CuI increase linearly with increasing ΔT, as described by the generic equation:

2 $${V}_{{\rm{out}}}={\rm{n}}({S}_{p}-{S}_{n}){\rm{\Delta }}T-I{R}_{int}$$

where *V*_out_ is the output voltage at the load resistance terminals to obtain the maximum power (P_out_), S_p_ and S_n_ are respectively the Seebeck coefficient for *p*- and *n*- semiconductor materials and n is the number of elements in series; *I* is the total current flow in the circuit and *R*_int_ the internal resistance of the TE element. For single element, the formula reverts to *V = S*_*n/p*_ *∆T − IR*_*int*_. The output power is described by the equation:

3 $${P}_{{\rm{out}}}=S{\rm{\Delta }}TI-{I}^{2}{R}_{{\rm{int}}}$$

Note that although comparable in transparency, CuI has four times the *P*_*out*_ of GZO (0.17 nW in GZO vs. 0.57 nW in CuI at 13 °C ΔT). Although GZO has a lower S value (−60 μVK^−1^) than that of CuI films (+206 μVK^−1^), GZO films show an electrical conductivity of one order of magnitude higher, hence, the PF value is similar for both materials (5×10^−4^ Wm^−1^ K^−2^). With the lowest measured *k* value for CuI of circa 0.64 Wm^−1^ K^−1^ we obtained ZT values of 0.22 at 300 K.

## Discussion

As evidenced in the above results, the fabrication of CuI thin films by the three different methods followed a thorough study of films in terms of their crystallography, morphology, UV-vis-NIR spectroscopy and thermoelectric properties. The optimisation of these films resulted in the first highly transparent thermoelectric *p*-*n* modules.

Structurally, in addition to a broader size distribution, solid iodination seems to favour the (220) oriented plane as opposed to the (111) in vapour iodination and thermal evaporation methods. One can attempt to conclude that, upon iodination, excess of iodine to a large extent forces the iodination of a copper film towards a preferential (220) diffraction plane. The vapour process is based on the surface chemisorption of vapour I_2_ when it reaches the Cu film as the reaction takes place favouring the (111) plane. Kinetically, this process strongly depends on the Cu film porosity and anchoring points (surface irregularities) where chemical adsorption starts. Solid iodination is based on a direct contact of an excess of solid I_2_ molecules with Cu films and thus less diffusion-limited. Therefore, this process is kinetically faster when in excess of I_2_ but slower in available time for structure rearrangements, originating finer grains with lower values of surface roughness as observed by SEM and AFM images. According to Shiojiri^17^, the iodine diffusion in copper has a constant rate (kp) parabolic with temperature and an activation energy of 3.9 kcal mol^−1^ for a pressure of 32 mTorr leading to the formation of a 1 µm film thickness in about 80 seconds. Although our films were formed at atmospheric pressure and at room temperature, a higher I^−^ diffusion rate is expected as in fact it is visually observed as the metal colour of Cu becomes a transparent film almost instantly upon contact with I_2_.

Some researchers^12,18^ argue that the (111) direction is not the most desired because iodide vacancies are enhanced as this is the most compact crystallographic plane of the zinc blend structure. Considering that iodine ionic radius (0.206 nm) is larger than Cu (0.074 nm), the (220) growth direction can better host the iodine atoms with less distortion to the networks as evidenced in Table 1 – the solid method shows a lower lattice strain value than vapour or thermal evaporation methods.

The overall performance of thermoelectric thin films critically depends on both crystal orientation and film nanostructure. These are often engineered as to improve electrical transport whilst at the same time aiming at introducing grain boundaries to increase phonon scattering. As all methods for CuI thin films preparation are very characteristic in terms of crystal orientation and nanostructure, conclusions can be drawn as to why the solid iodination method delivered higher PF values compared to thermal and vapour methods. The thermal evaporation method predominantly favours the (111) orientation, which is a rather compact and crystal dense face. Although AFM reveals thermal evaporated CuI thin films less rough with lower grain sizes, the highly packed vertical (111) growth supports a good flow of phonons and thus revealing a lower Seebeck coefficient, but at the same time allowing an excess of iodine vacancies due to ionic radii mismatch and incomplete non-stoichiometric crystallisation (characteristic of physical vapour deposition (PVD)) and hence compromising electrical conductivity. The advantage of grain growing a (220) parallel face over (111) oriented crystals is that the former is expected to show less crystal density and to yield improved electrical performance, as reported by Zheng *et al*.^12^ in CuI films and Abutaha *et al*.^19^ in AZO thin films. While a parallel and less dense face growth allows for greater diffusion of free electrons across planes – a flow with a direction parallel to both electrodes; samples with preferential (220) faces will also reveal more phonon scattering across the less dense, porous γ-CuI lattice. A mixture of (111) and (220) orientations in solid method results in further phonon scattering whilst the predominant (220) orientation significantly contributes to increased free electron conduction.

Optically, samples prepared by vapour iodination method show a lower specular transmittance in the visible range of the spectrum owing to their surface morphology as this method originates columnar-like morphologies with high-depth-ratios visible in SEM images and AFM maps – resulting in larger yields of scattered light.

Nevertheless, optical band gap transitions of CuI are dictated by a strong absorption at the direct band gap where free excitons recombine and copper vacancies are the dominant defect contributing to band transport. In contrast to copper oxides, the *p* valance state of iodine is expected to delocalize the electron-holes nearer the top of valance band leading to a split off band seen in the transmittance spectra and localized at 3.65 eV in agreement with literature findings^5^. The acceptor level above the band gap enhances hole mobility by providing with electron hole delocalisation at the top of the valence band. It is confirmed that the *p*-type conductance originating from Cu vacancies and other defects, such as Cu interstitials and iodine vacancies, are known to impede hole carriers^20^. Consequently, it is essential to maintain stoichiometry and to keep defects to a minimum. PVD techniques are known to be non-stoichiometric and as such thermal evaporation was quickly ruled out as samples prepared by this method have lower values of carrier concentration *N* and mobility *µ*. Supplementary Table 4 summarises hole concentration and mobility per method.

Overall, this study demonstrates solid iodination of Cu films to be the method which collectively performs better in both thermoelectric properties and optical transparency.

Several authors^7,18^ have reported annealing temperatures to influence CuI thermoelectric properties. We verified that annealing in vacuum post-CuI formation via solid method not only enhanced the Seebeck coefficient but also resulted in increased electrical resistance (100-fold increment) and therefore the PF became much lower (1.8×10^−5^ Wm^−1^ K^−2^). These findings are consistent with the idea of a change in the scattering mechanism of free electrons by introducing iodine vacancies as phonon scattering centres – hence the linear dependence of *S* on *T*. At annealing temperatures higher than 300 °C in vacuum, CuI samples resulted in depleted films with an overloaded resistance reading owing to a likely enhancement of the Cu acceptor level due to the weekly-bonded iodine being more prone to sublimate from the copper phase at higher temperatures and thus generating an excess of surface-level deficiencies and vacancies. Furthermore, it is known that CuI assumes a variety of polymorphs at varying temperature and pressure ranges^5^: at atmospheric pressure the rock salt (α-CuI) phase is shown above 673 K, the wurtzite phase (β-CuI) for temperatures in the range of 643–673 K, and the zinc blend below 673 K; whilst with an hydrostatic pressure of 1.4 GPa at ambient temperature α-CuI phase changes to rhombohedral structure and at 4 GPa it assumes an orthorhombic structure. As such, phase transformations of γ-CuI may occur during the annealing process in vacuum towards more or less favourable carrier conduction resulting in lower thermoelectric and optical properties.

**Figure 6:**
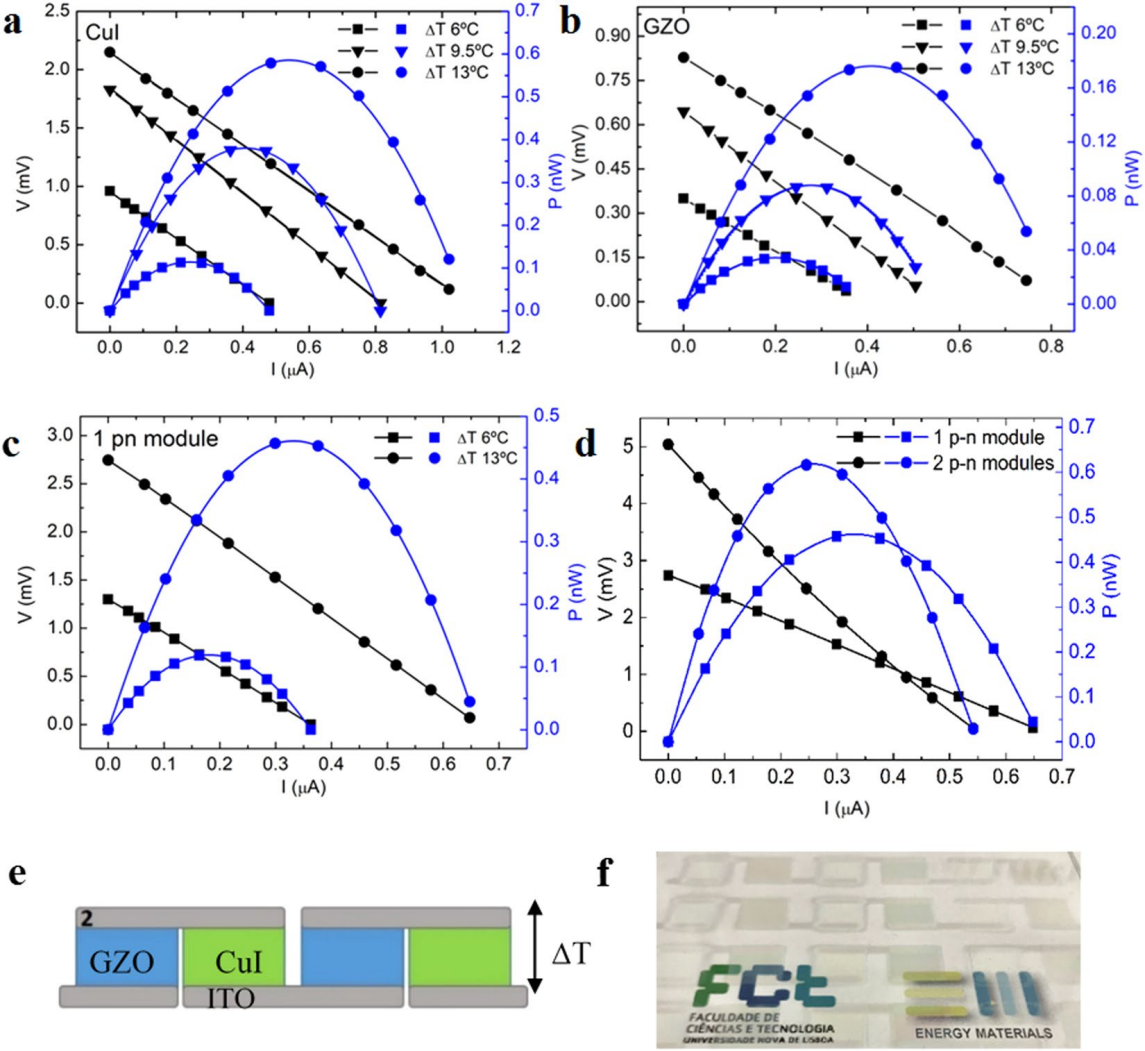
I-V and P-V curves of single TE elements of CuI (**a**) and GZO (**b**). Combining the two elements to produce 1 (**c**) and 2 *p-n* modules (**d**); Schematic of two GZO and CuI *p*-*n* modules electrically connected in series and thermally in parallel (**e**) with a photograph of the equivalent transparent two modules (**f**).

I-V and P-V curves of a single TE module (solid method CuI/GZO) reveal a generated power output of 0.46 nW at ∆T = 13 °C which is in accordance with equations (2) and (3) for the sum of the elements and accounting for internal and interconnection resistance. V_oc_ and I_sc_ values linearly depend on Δ*T*, resulting in a four-fold increase in the value of generated power P_out_ in line with equation (3), as shown in Fig. 6c for 1 *p*-*n* module (from 0.12 to 0.46 nW at a Δ*T* = 7).

The total power density of the device can be increased by connecting several *p-n* modules in series as evidenced by the two module P-V curve. Here, I_sc_ values are expected to be equal; however, the module with the lowest current at zero voltage will dominate the in-series device as demonstrated in the one vs. two module curves. This is reasonable when accounting for minor internal and interconnection resistances. The V_oc_ value is rightly doubled and consequently the maximum power output should too at constant values of I_sc_.

Nonetheless, although a double module power output is still small for most planar geometry applications, assuming no film degeneracies causing higher resistance between modules, an equivalent of 7 CuI/GZO modules can yield a comparable power output of a single planar Sb_2_Te_3_/Bi_2_Te_3_ thermocouple for a maximum output of 2 nW at a ∆T = 10 °C^21^.

In conclusion, highly transparent *p*-type CuI thin films can be fabricated via the facile process of solid iodination of a copper film to achieve a PF of 4.7×10^−4^ Wm^−1^ K^−2^ and a maximum ZT value of 0.22, a thousand times higher than state of the art *p*-type transparent materials such as CuAlO^22^. We have demonstrated the first ever prototype of a fully transparent TE module and we hope this study incites further developments in the field of transparent devices and thermoelectricity. CuI has proven with great TE potential and easily adapted to transparent thin film technology.

## Experimental Details

The iodination of Cu thin films, obtained by resistive thermal evaporation on corning glass substrates with thicknesses ranging from 20 to 150 nm, was performed by vapour and solid phase methods. Each Cu thin film was placed in a sealed box with the Cu layer facing upwards. In vapour iodination, sublimated iodine was introduced into the box via N_2_ gas as an inert carrier and the copper film iodinated in an iodine oxygen-free controlled atmosphere. The optimisation of the process followed by varying copper film thicknesses from 20 to 100 nm, and iodine exposure times from 60 to 240 minutes.

In solid iodination, the iodination occurred in a sealed box which was purged with N_2_ prior and after solid particles of iodine were placed on top of the Cu thin film. The reaction time varied from 30 to 120 minutes to allow for sample optimisation.

The iodination process occurs according to the following equation:

4 $$2Cu+{I}_{2(g/s)}\to 2CuI$$

**Figure 7:**
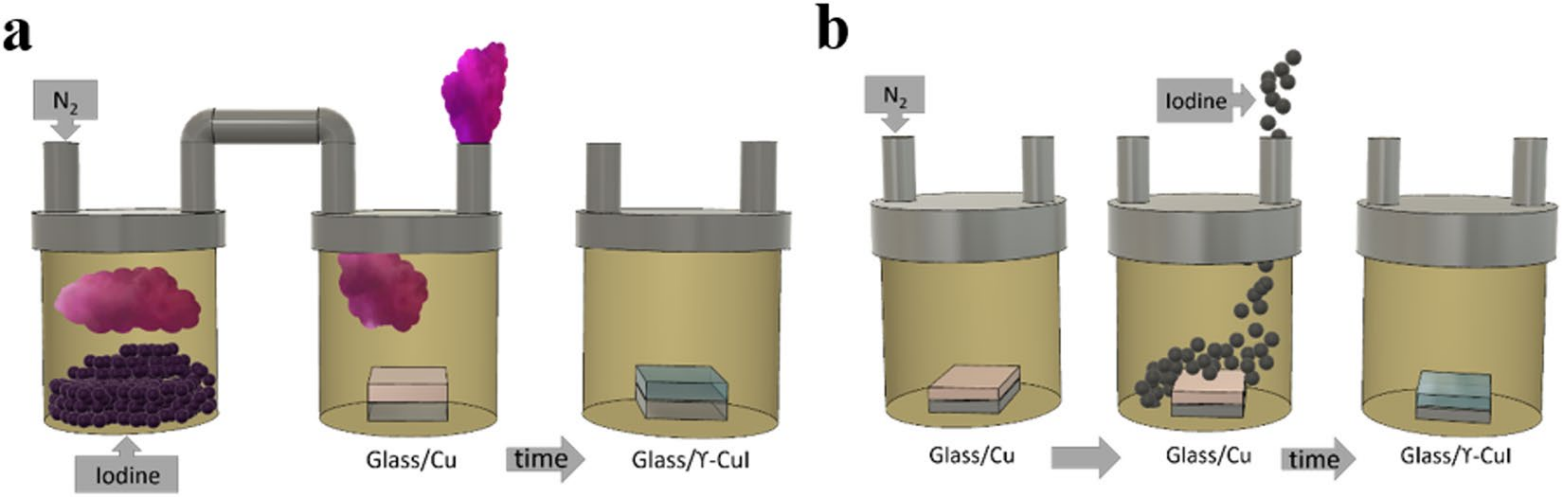
Schematic of the iodination process: (**a**) vapour phase; (**b**) solid phase.

In both cases, reactions were carried out at room temperature with the iodination of the Cu film being visible at the naked eye as the mirror-like brown Cu thin film gradually becomes a transparent γ-CuI thin film (Fig. 7). A summary of the initial Cu film thicknesses chosen and relevant properties arising from solid and vapour iodination reactions is represented in Supplemetary Tables 2 and 3, respectively.

γ-CuI thin films were also obtained by resistive thermal evaporation (3.0×10^−7^ hPa, 1 nm.s^−1^) of CuI powder (purity 99.9% Sigma Aldrich®). Optimisation of this involved varying film thicknesses in the range of 50 to 310 nm, detailed in Supplementary Table 1.

**Figure 8:**
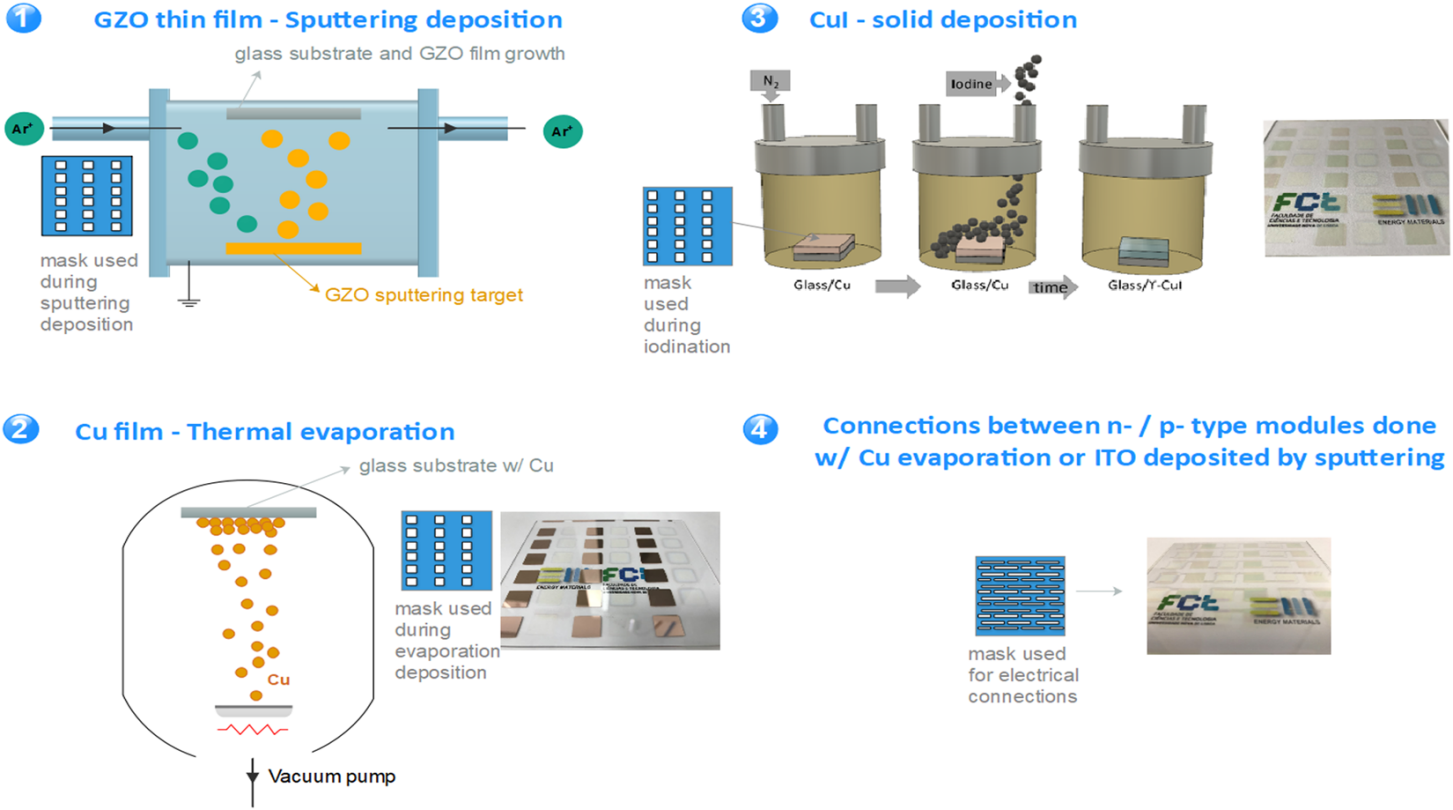
A schematic representation of the fabrication process of the first fully transparent TEG made of *p*-type CuI and *n*-type GZO elements electrically connected in-series and thermally in parallel.

Figure 8 illustrates the fabrication of transparent TEG modules: (1) in a 10×10 cm^2^ corning glass, a mask with 1.5×1.5 cm^2^ squares vertically aligned is applied where GZO thin films (361 nm in thickness) as *n*-type material are deposited by RF magnetron sputtering using a GZO (ZnO:G 95:5) target; Subsequently, another mask is applied with 1.5×1.5 cm^2^ squares parallel to the previously deposited thin films is applied where Cu metal is deposited by resistive thermal evaporation to the optimised Cu thickness (2); (3) solid iodine is then applied to the Cu surface via the process described in Fig. 6; (4) finally, anoher mask is applied over the two collumns where ITO thin films (150 nm in thickness) as in-series electrical contacts were deposited by RF sputtering using an ITO target. Sputtering targets were supplied by Super Conductor Materins Inc and Plasmaterials for ITO and GZO (25% Ga), respectively. Electrical properties of sputtered thin films were optimised by ajusting RF power in the range of 100–150 W and working pressure between 1.2–2.0 mTorr, versus deposition time.

Transmittance and reflectance spectra of CuI thin films on corning glass substrates were recorded on a Jasco V-770 double beam spectrophotometer in the wavelength range from 200 nm to 1500 nm.

**Figure 9:**
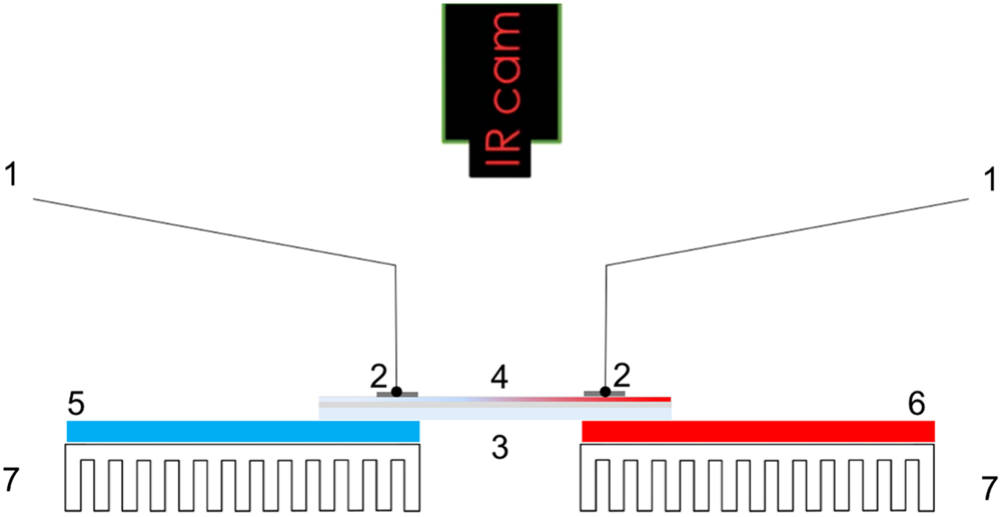
A schematic of the cross-sectional view of the home-made apparatus for the Seebeck measurements of thin films. (1) Nano-voltmeter probes; (2) Contacts on thin film; (3) Glass substrate; (4) Thermoelectric thin film; (5) Cold Peltier; (6); Hot Peltier; and (7) heat-sink.

Measurements of seebeck coefficient, electrical conductivity, and resistance were measured in a homemade apparatus, illustrated in Fig. 9.

Samples were placed on top of two Peltier modules (TEC1-12707) as detailed in our previous work^1^. Bare Conductive® Carbon ink or ITO contacts separated by 1 cm were deposited on the film. A thermal camera (FLIR A310) was used to measure the temperature gradient between contacts which in turn was varied by the heating and cooling of the two Peltier elements under the sample. An equal length of CuI film was over both cold and hot Peltier elements (connected to different power sources) to avoid an unbalanced cooling/heating of the sample. The potential difference was measured using a nanovoltmeter Agilent 34420 A.

Measurements of carrier Hall mobility (*µ*), electrical conductivity (σ) and concentration (*N*) were measured on a Hall-effect measurement system Bio Rad HL 5500 using the van der Pauw configuration.

The thermal conductivities were measured at room-temperature using the Linseis thin film laserflash analyzer as reported elsewhere^23^. In brief, the system utilizes the nanosecond transient thermoreflectance (TTR) method, in which a sample coated with 20/200 nm Ti/Au metallic layer is heated by a 8 ns pump laser pulse (Nd:YAG, 1064 nm, 10 Hz). A 463 nm diode pumped solid-state laser is used to probe the reflectance of the heated gold film, directly proportional to the transient temperature change on the film surface. The thermoreflectance signal is averaged over 80 pulses and then fitted to a one-dimensional transmission line model of the multilayer system, in which bulk thermophysical properties of the constituent materials were assumed for extracting the sample thermal conductivities^24^. Twelve positions along the sample surface were analysed in order to average out spatial variation and produce confidence intervals for the results.

Raman spectroscopy was carried out by a Witec Alpha 300 confocal RAS with a 532 nm Argon Laser at 0.5 mW of power. Atomic Force Microscopy (AFM) was performed with the same equipment operating in AC Mode with lateral resolution down to 1 nm and depth resolution below 0.3 nm. The samples were scanned under a probe with Al reflex coating and resonant frequency of 75 kHz at a constant force of of 2.8 N/m.

Surface Morphology and elementary composition of the samples were characterized by a scanning electron microscope (SEM) Hitachi S2400 with Bruker light elements EDS (for EDS sepctra and corresponding maps see Supplementary Figure 2). Samples were placed in the sample holder using a carbon ribbon and then covered with a thin layer of gold-palladium alloy. Sample thicknesses were measured in a Perfilometer KLA Tencor D-600.

X-ray crystallography analysis were performed in a X-ray diffractometer (XRD) PANalytical X’Pert PRO equipped with an X’Celerator detector using Cu Kα radiation at 45 kV and 40 mA, in a Bragg–Brentano configuration and the XRD patterns were collected over the angular 2θ range 10–90° with a scanning step of 0.03°.

## Electronic supplementary material

Supplementary Information
