# Supplementary material for: CuI p-type thin films for highly transparent thermoelectric p-n modules
Source: Sci Rep. 2018 May 2;8:6867. doi: 10.1038/s41598-018-25106-3 (PMC5932081; doi:10.1038/s41598-018-25106-3)
Supplement: Supplementary file 1 — Supplementary Information [file 41598_2018_25106_MOESM1_ESM.pdf]

SUPPLEMENTARY INFORMATION

CuI p-type thin films to highly transparent thermoelectric p-n modules

Bruno Miguel Morais Faustino[1]*, Diogo Gomes[1], Jaime Faria[1], Taneli Juntunen[2], Guilherme Gaspar[1], Catarina Bianchi[1], António Almeida[1], Ana Marques[1], Ilkka Tittonen[2] and Isabel Ferreira[1]

[1]CENIMAT/I3N, Departamento de Ciência dos Materiais, Faculdade de Ciências e Tecnologia, Universidade Nova de Lisboa, Caparica 2829-516, Portugal.

[2]Department of Electronics and Nanoengineering, Aalto University, P.O. Box 13500, FI-00076 Aalto, Finland

E-mail: *bm.faustino@fct.unl.pt

Supplementary Table 1: Variation of the thermoelectric properties of CuI thermally evaporated with film thicknesses

Material	Thickness (nm)	Conductivity ($\Omega^{-1}m^{-1}$)	Seebeck (μV/K)	PF (W/m.K^2)
**CuI Thermal Evaporated**	80	1.3×10^3	158.1	3.3×10^{-5}
	50	3.7×10^3	161.3	9.6×10^{-5}
	310	2.4×10^2	189.6	8.5×10^{-6}

Supplementary Table 2: Thermoelectric properties of CuI films deposited by solid method

Thickness Cu (nm)	Thickness CuI (nm)	Conductivity ($\Omega^{-1}m^{-1}$)	Seebeck (μV/K)	PF (W/m.K^2)
**72**	302	2.8×10^3	156.0	6.7×10^{-5}
**92**	333	2.0×10^3	206.0	8.5×10^{-5}
**85**	325	1.9×10^3	145.0	4.0×10^{-5}
**65**	287	1.1×10^4	206.1	4.9×10^{-4}
**134**	465	3.5×10^3	207.3	1.5×10^{-4}

Supplementary Table 3: Thermoelectric Properties of CuI films deposited by vapour method as a function of the initial Cu thickness.

Thickness Cu (nm)	Thickness CuI (nm)	Conductivity ($\Omega^{-1}m^{-1}$)	Seebeck (μV/K)	PF (W/m.K^2)
**20**	68	1.7×10^3	88.4	1.3×10^{-5}
**10**	66	1.2×10^3	103.6	1.3×10^{-5}
**50**	269	4.0×10^2	182.3	1.3×10^{-5}
**80**	302	7.4×10^2	158.1	1.9×10^{-5}
**150**	581	1.3×10^2	189.6	4.6×10^{-6}

Supplementary Table 4: Thermoelectric properties of CuI films produced by all methods

Method	Thickness (nm)	Conductivity ($\Omega^{-1}m^{-1}$)	Seebeck ($\mu V/K$)	PF ($W/m.K^2$)	Hole Density (cm^{-3})	Hall Mobility ($cm^2V^{-1}s^{-1}$)
**Solid**	287	1.1×10^4	206.0	$4.7x10^{-4}$	1.68×10^{20}	4.1
**Vapour**	302	7.4×10^2	158.1	$1.9x10^{-5}$	2.01×10^{19}	2.3
**T.Evap.**	50	3.7×10^3	161.3	$9.6x10^{-5}$	5.93×10^{19}	3.9

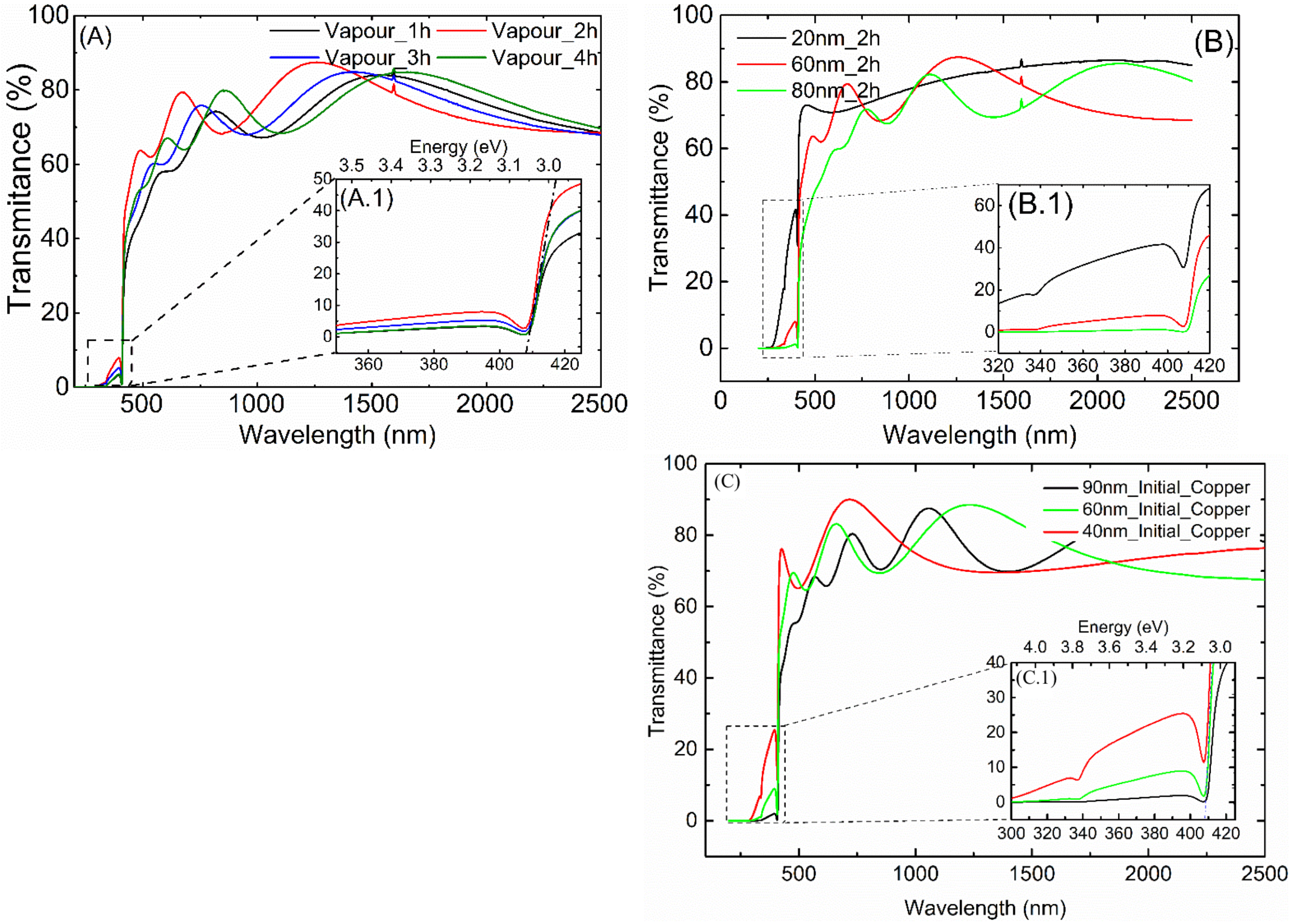

Supplementary Figure 1 – (A) shows specular transmittance of vapour deposited films for different times of iodination. Inset (A.1) zoom-in at 350 to 430 nm wavelength range and extrapolated band gap energy. (B) shows the variance of transmittance when changing the initial copper thickness for a vapour iodination time of 2h. And (B.1) represents the zoom-in at 320 to 420 nm wavelength range. (C) shows the variation of specular transmittance with initial copper thickness in solid method; The inset (C.1) is a zoom-in at 300-420 nm.

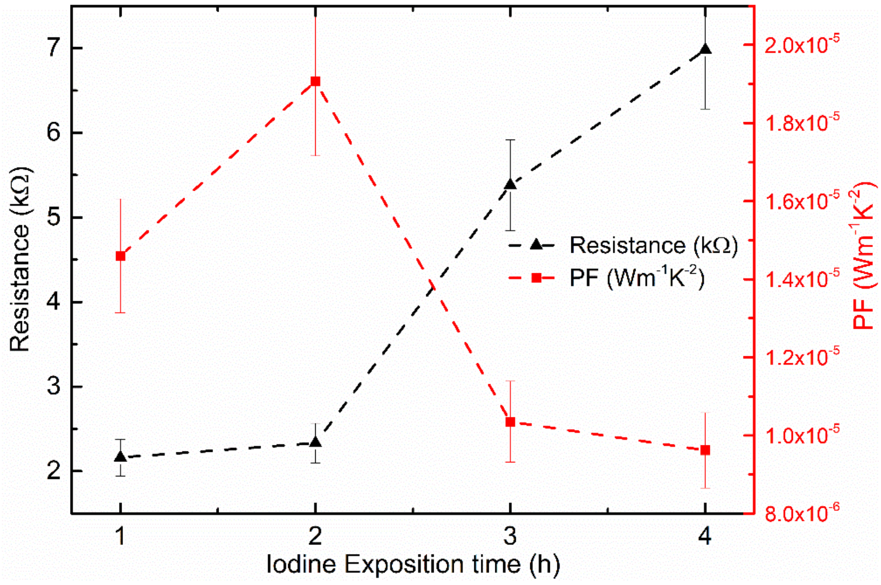

Supplementary Figure 2 – CuI Vapour Iodinated Resistance and Power Factor over iodine exposition time.

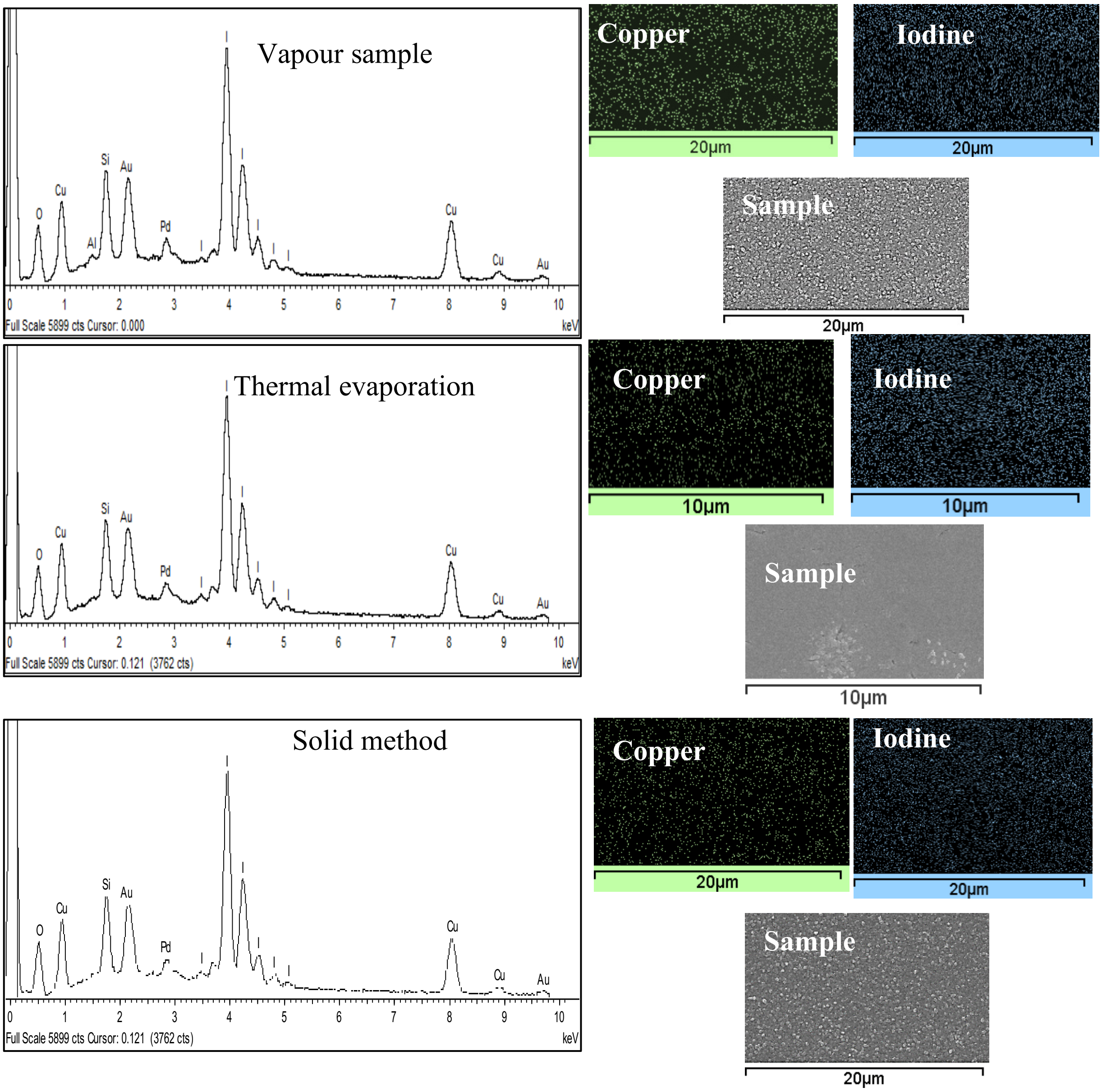

Supplementary Figure 2 – The EDS spectra and respective elements mapping for samples produced by the three different methods studied. Relevant peaks are assigned to copper and iodine. All others are either from SEM coating or the substrate.

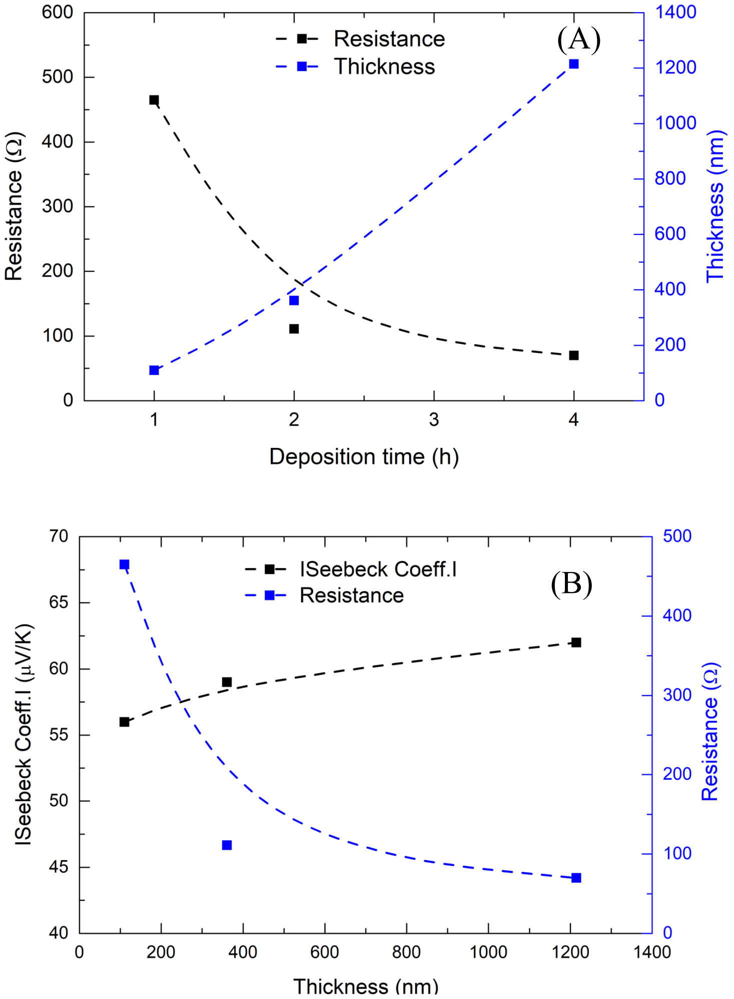

Supplementary Figure 3 – Gallium doped-zinc oxide (5%) deposited by RF magnetron sputtering was optimised at 130W of RF power and 1.8 mTorr of working pressure versus the deposition time in order to: (A) minimise resistance at the smallest thickness possible whilst monitoring the Seebeck coefficient as a function of thickness (B).
